# Comorbidities and outcomes of patients with chronic myeloid leukemia treated with tyrosine kinase inhibitors: a real-world, nationwide, retrospective study from Hungary

**DOI:** 10.3389/pore.2024.1611497

**Published:** 2024-02-20

**Authors:** Peter Batar, Hussain Alizadeh, Gyorgy Rokszin, Zsolt Abonyi-Toth, Judit Demeter

**Affiliations:** ^1^ Department of Hematology, Faculty of Medicine, University of Debrecen, Debrecen, Hungary; ^2^ 1st Department of Medicine, Division of Haematology, Clinical Center, Medical School, University of Pecs, Pecs, Hungary; ^3^ RxTarget Ltd., Szolnok, Hungary; ^4^ Department of Biostatistics, University of Veterinary Medicine, Budapest, Hungary; ^5^ Department of Internal Medicine and Oncology, Faculty of Medicine, Semmelweis University, Budapest, Hungary

**Keywords:** chronic myeloid leukemia, real-word evidence, tyrosine kinase inhibitors, treatment outcome, overall survival

## Abstract

**Purpose:** This study aimed to provide real-world evidence on the characteristics, treatment patterns, and outcomes of patients with chronic myeloid leukemia (CML) receiving tyrosine kinase inhibitor (TKI) treatment in Hungary between 2011 and 2019.

**Patients and methods:** This nationwide, retrospective study included patients who were newly diagnosed with CML in Hungarian clinical practice between January 2011 and December 2019. The analysis was based on the reimbursed prescription claims for imatinib, bosutinib, dasatinib, nilotinib, or ponatinib with the ICD-10 code C9210 in a public pharmacy between January 2009 and December 2019 using data from the National Health Insurance Fund (NHIF) of Hungary. CML incidence and prevalence, TKI treatment patterns, comorbidities, and overall survival (OS) were examined.

**Results:** Between 2011 and 2019, altogether 1,407 patients were diagnosed with CML, with an annual average of 156 patients. The number of patients newly initiating first-line TKI therapy for CML significantly increased between 2011 and 2019 (2011: *n* = 136 vs. 2019: *n* = 191; *p* = 0.0043). Nilotinib was typically prescribed for younger patients (≤64 years), while older patients (≥65 years) mostly received imatinib. The most common comorbidity of CML patients was hypertension, and the proportion of patients with other malignancies was relatively high in all treatment groups. 5-year OS was 77.1% during the whole study period. Patients initiating first-line TKI treatment for CML in 2015 had significantly better 4-year OS compared to those starting treatment in 2011 (82.4% vs. 73.5%, respectively, (HR 0.53 (95%CI 0.32–0.87) *p* = 0.0118).

**Conclusion:** This study is the first to provide insights into the characteristics, treatment patterns, and outcomes of CML patients treated with TKIs in Hungarian clinical practice between 2011 and 2019. We found slightly lower OS rates compared to other European countries, however, there was a statistically significant improvement in 4-year OS during the study period. The management of CML was in line with international guidelines and recommendations.

## Introduction

Chronic myeloid leukemia (CML) accounts for 10%–15% of all leukemia cases, with an estimated incidence of 0.7–1.8/100,000 persons worldwide [1]. The course of CML can be divided into chronic, accelerated and blastic phases, and most patients are diagnosed during the chronic phase when the disease is often asymptomatic or very few symptoms are present [2].

The prognosis of CML has dramatically improved over the past two decades, due to the introduction of tyrosine kinase inhibitors (TKI) [2]. The development of imatinib, a first-generation TKI, represented the first real breakthrough in the treatment of CML [3]. Since then, four further TKIs have been added to the therapeutic armamentarium: the second-generation TKIs dasatinib, nilotinib, bosutinib, and the third-generation TKI ponatinib [4]. As a result of targeted therapy, in developed countries, the life expectancy of CML patients diagnosed in the chronic phase and receiving TKI therapy is very close to that of the age-matched general population, with 5-year survival rates of around 90% [5–7].

The 2020 guidelines of the European LeukemiaNet (ELN) recommend TKI for the first-line treatment of CML [5]. Imatinib is approved by the United States (U.S.) Food and Drug Administration (FDA) and the European Medicines Agency (EMA) for first line treatment while dasatinib, nilotinib, and bosutinib are registered in the first-, second- and further-line settings, and the third-generation TKI ponatinib is recommended for treatment beyond second-line. The treatment of CML requires close monitoring to allow for the timely detection of relapses or intolerable toxicities. In these cases, the ELN 2020 recommends the continuation of therapy with another TKI for up to four lines, with the careful consideration of patient-related factors including age, comorbidities, and previous TKI-related toxicities. As TKIs have different contraindications, early and late toxicity profiles, appropriate patient selection is of utmost importance to ensure good tolerability and optimize treatment outcomes.

The first registered TKI for treatment of CML was imatinib which was approved in 2001. In Hungary, nilotinib was approved for the first-line treatment of CML in 2014, which was soon followed by the approval of dasatinib in 2015. Bosutinib gained approval for second and subsequent lines of therapy in 2013 and for first line treatment of CML in 2018, while ponatinib is currently the only effective option in the case of T315L resistance mutations, approved for second- and subsequent treatment lines since 2013. The reimbursement of the TKIs may influence the application of treatment options.

Although clinical trials have consistently demonstrated the survival benefits and good tolerability of TKIs among patients with CML, there is a need for real-world evidence to gain more insights into the performance of these drugs in unselected patient populations encountered in routine clinical practice [8]. Several studies have reported real-world evidence on the characteristics, treatment patterns, and outcomes of CML patients treated with TKIs [9–19], however, no nationwide data have been published so far from Hungary in this regard.

Therefore, the aim of the current study was to examine the characteristics, outcomes, and survival of CML patients receiving TKI treatment in real-world Hungarian clinical practice between 2011 and 2019, focusing on treatment patterns, comorbidity profiles, and overall survival (OS).

## Material and methods

This was a nationwide, retrospective study which included patients newly diagnosed with CML in Hungarian clinical practice between January 2011 and July 2020 who had at least one reimbursed prescription claim for imatinib, bosutinib, dasatinib, nilotinib, or ponatinib with the ICD-10 code C9210 in a public pharmacy between January 2009 and June 2020 based on the database of the National Health Insurance Fund of Hungary (NHIF). The NHIF contains patient ID and ICD-10 code information about all in- and out-patient visits as well as prescriptions of reimbursed drugs in Hungary. The NHIF covers the whole Hungarian population, therefore it served as a comprehensive data source for our nationwide study. Of note, the diagnosis of CML was established merely in a deductive way based on prescription claims for TKIs; no information on cytogenetics or molecular genetics were available. The requested data from NHIF covered the patient data from January 2011 and July 2020 however to be able to demonstrate whole year analyses this work includes data from the period of January 2011 and December 2019.

The incidence and prevalence of CML were examined between January 2011 and December 2019. The 2009-2010 period served as a screening period for the identification of newly diagnosed CML patients and was not included in the analysis. Incidence and prevalence were examined in the whole patient population as well as according to age (0–64 vs. >65 years), sex, and line of therapy.

Treatment decisions regarding the choice of first-line TKI and change of therapy were at the discretion of the treating physician and were carried out according to EMA label recommendations. Patients already receiving ongoing imatinib therapy in 2009 were considered first-line. A new line of therapy was recorded if one of the following two scenarios occurred: i) a new TKI was initiated at least 60 days after the first TKI initiation; ii) a new TKI was initiated which was not claimed within 30 days prior to the previous TKI. Therefore, if a patient was switched from one TKI to another within 60 days, both TKIs were registered as first-line therapy (e.g., in the case of an imatinib-nilotinib-nilotinib-nilotinib sequence if nilotinib was initiated within 60 days after imatinib), and the recurrence of a TKI after a previous change of treatment line was not regarded as a new change of line (e.g., in the case of an imatinib-imatinib-imatinib-nilotinib-imatinib-imatinib-imatinib sequence). In summary, patients may have appeared with two different TKIs within the same treatment line. As a result of this methodology, the sum of annual patient numbers for a given TKI may have exceeded the total number of CML patients in the same year.

Comorbidities were examined 365 days prior to the initiation of therapy and were defined based on conditions included in the Charlson Comorbidity Index (CCI) [20] for the period between January 2011 to June 2020. The observation period for the analysis of comorbidities was extended until June 2020 to ensure that comorbidities affecting a low number of patients were also included in the analysis as the NHIF does not provide data with patient numbers below 10. Patients were classified as having a comorbidity based on one ICD-10 code record in in-patient care or an ICD-10 code record in out-patient care and at least one record of the same ICD-10 code in in- or out-patient care within more than 30 but fewer than 360 days following the first record. Of note, the identification of comorbidities was solely based on ICD-10 code records, and not on clinical findings or cardiology registries (Supplementary Table S1). Comorbidities were examined in the whole patient population and according to type of TKI and line of therapy.

For patients eligible for inclusion, all types of care recorded in the NHIF were queried including in- and out-patient visits, prescription claims, CT or MRI, and hemodialysis irrespective of their association with CML.

## Statistics

Overall survival (OS), defined as the period between the initiation of TKI therapy for CML and death, was estimated using the Kaplan-Meier method between 1 January 2011 and 31 December 2019. 4-year OS rates were calculated separately for patients initiating first-line TKI therapy in 2011 and 2015.

We used Cox regression to analyse the change of the survival in time. We corrected for the differences in the baseline hazard by gender and age group (0–64, 65-).

We analysed the chage of age group distribution in time using binomial logistic regression. We used linear regression to calculate the annual trend for the number of new cases/100.000 patient years.

The study protocol was approved by Hungarian National Institute of Pharmacy and Nutrition, and by Medical Research Council Ethical Committee (IV/5969-1/2020/EKU, IV/210-1/2021/EKU) and was carried out in accordance with the 1975 Declaration of Helsinki, as revised in 2000, and Good Clinical Practice guidelines. Data collection and analysis was performed on anonymized and aggregated data.

## Results

Between 2011 and 2019, altogether 1,407 patients were newly initiated on TKI therapy for CML, with an annual average of 156 patients (mean age: 56 years, 52% male). The incidence of CML varied between 1.16/100,000 in 2012 and 1.96/100,000 in 2019. There was a statistically significant increase in the number of patients newly initiating first-line therapy for CML between 2011 and 2019 (2011: *n* = 136 vs. 2019: *n* = 191; *p* = 0.0043) (Table 1). The number of patients initiating second-line therapy for CML remained constant during the observation period (2011: *n* = 79 vs. 2019: *n* = 73; *p* = 0.9875), with an annual average of 74.

**TABLE 1:** Incidence and number of patients initiating first-line treatment for CML according to age and sex between 2011 and 2019 in Hungary.

	2011–2019	2011	2012	2013	2014	2015	2016	2017	2018	2019
new 1L incidence/100,000		1.36	1.16	1.46	1.72	1.55	1.71	1.66	1.70	1.96
new 1L (n)	1,407	136	116	144	170	153	168	163	166	191
male	52%	54%	49%	53%	51%	52%	48%	58%	59%	46%
female	48%	46%	51%	47%	49%	48%	52%	42%	41%	54%
mean age (years) (SD, years)	56.13 (±17.15)	55.35 (±16.42)	55.15 (±16.69)	56.25 (±17.28)	55.83 (±16.87)	56.02 (±18.63)	56.46 (±16.35)	57.19 (±17.07)	55.40 (±18.41)	57.57 (±16.67)

CML, chronic myeloid leukemia; SD, standard deviation; 1L, first line.

**FIGURE 1:**
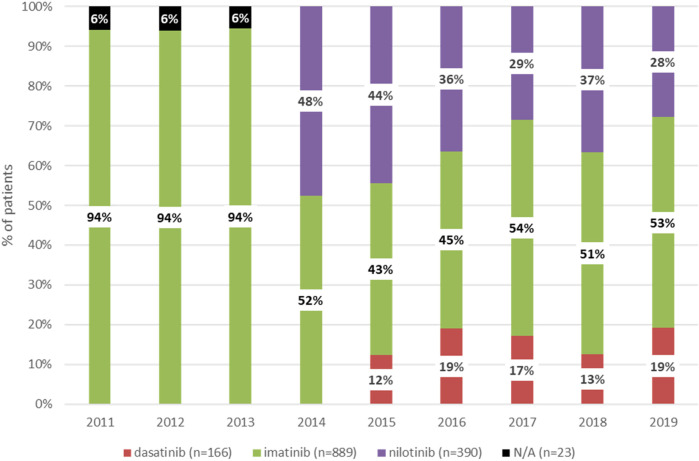
Distribution of patients newly receiving first-line treatment for CML between 2011 and 2019 according to type of TKI. CML, chronic myeloid leukemia; TKI, tyrosine kinase inhibitor. N/A, not applicable (NHIF does not provide data with patient numbers below 10).

Figure 1 shows the distribution of patients according to the type of TKI in the first-line setting between 2011 and 2019. Between 2011 and 2013, all patients newly diagnosed with CML received imatinib as first-line treatment. Nilotinib and dasatinib were registered for first-line treatment in Hungary in 2014 and 2015, respectively. Therefore, since 2015, three TKIs have been available as first-line treatment options in Hungary (imatinib, nilotinib, and dasatinib). The proportion of patients receiving dasatinib remained constant between 2015 and 2019, the proportion of those treated with imatinib increased from 43% to 53%, and the proportion of patients receiving nilotinib decreased from 44% to 28%.

**FIGURE 2:**
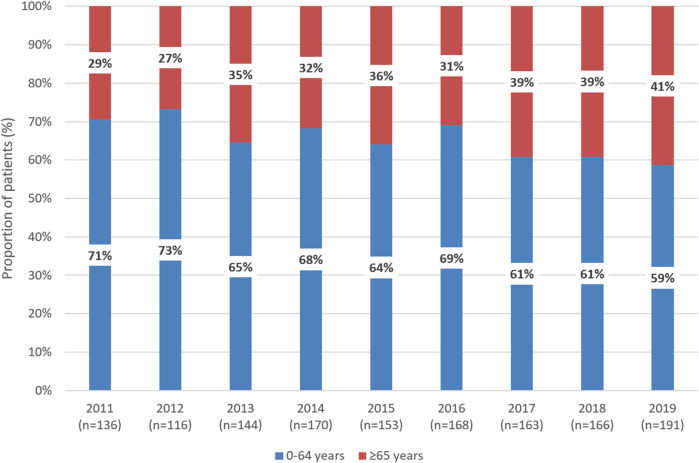
Distribution of patients receiving first-line TKI treatment for CML according to age 0–64 years vs. 65 years between 2011 and 2019 in Hungary. TKI, tyrosine kinase inhibitor; CML, chronic myeloid leukemia.

In 2011 and 2019, 29% and 41% of patients newly initiating first-line therapy for CML were 65 years or older, respectively (Figure 2). Among patients treated with imatinib, the proportion of patients aged 65 years or older significantly increased from 30% in 2011 to 50% in 2019 (*p* = 0.0035). The same trend was observed with dasatinib between 2015 and 2019 (10% vs. 39%; *p* = 0.0165). In contrast, the proportion of patients aged 65 years or older remained constant among those treated with nilotinib between 2014 and 2019 (Supplementary Figure S1). Among patients aged 65 years or older, the proportion of those receiving nilotinib decreased from 35% to 17% between 2015 and 2019, while the proportion of patients treated with imatinib increased from 32% to 65% (Supplementary Figure S2).

### Comorbidities of CML patients

In our study, 53.5% of all CML patients had at least one comorbidity at treatment initiation. In all treatment lines combined, the most common comorbidity of CML patients was hypertension. The proportion of patients with comorbidities was the highest among those receiving bosutinib (60.5%), and the lowest among patients treated with nilotinib (46.5%) (Supplementary Table S2).

At first-line treatment initiation, patients treated with nilotinib had the lowest number of comorbidities (41.6%), while those receiving imatinib (58.6%) had the highest. The proportion of patients with “Other malignancies” was relatively high in all treatment groups: in total, 159 patients had “Other malignancies,” of whom 108 were treated with imatinib, 31 received nilotinib, and 20 received dasatinib treatment (Table 2).

**TABLE 2:** Comorbidities among patients receiving dasatinib, imatinib, or nilotinib for CML in the first-line setting between 2011 and 2019.

	All TKI (*n* = 1403)	dasatinib (*n* = 161)	imatinib (*n* = 865)	nilotinib (*n* = 377)
Hypertension	511 (36.4%)	57 (35.4%)	345 (39.9%)	109 (28.9%)
Metabolic disorders	267 (19.0%)	34 (21.1%)	179 (20.7%)	54 (14.3%)
Diabetes mellitus	208 (14.8%)	31 (19.3%)	138 (16.0%)	39 (10.3%)
Ischemic heart disease	187 (13.3%)	17 (10.6%)	130 (15.0%)	40 (10.6%)
Other cancer	159 (11.3%)	20 (12.4%)	108 (12.5%)	31 (8.2%)
Pulmonary disease (lower tract)	120 (8.6%)	14 (8.7%)	86 (9.9%)	20 (5.3%)
Cerebral vascular accident	105 (7.5%)	10 (6.2%)	73 (8.4%)	22 (5.8%)
Arrythmiac condition (including AF)	94 (6.7%)	13 (8.1%)	60 (6.9%)	21 (5.6%)
Peripheral vascular disease	82 (5.8%)	10 (6.2%)	55 (6.4%)	17 (4.5%)
Congestive heart failure	70 (5.0%)	N/A^a^	49 (5.7%)	12 (3.2%)
**TOTAL WITH COMORBIDITIES**	**750 (53.5%)**	**86 (53.4%)**	**507 (58.6%)**	**157 (41.6%)**

CML: chronic myeloid leukemia, AF: atrial fibrillation.

^a^ Groups fewer than 10 are automatically cancelled by the National Health Insurance Fund (NHIF) due to data protection reasons.

**FIGURE 3:**
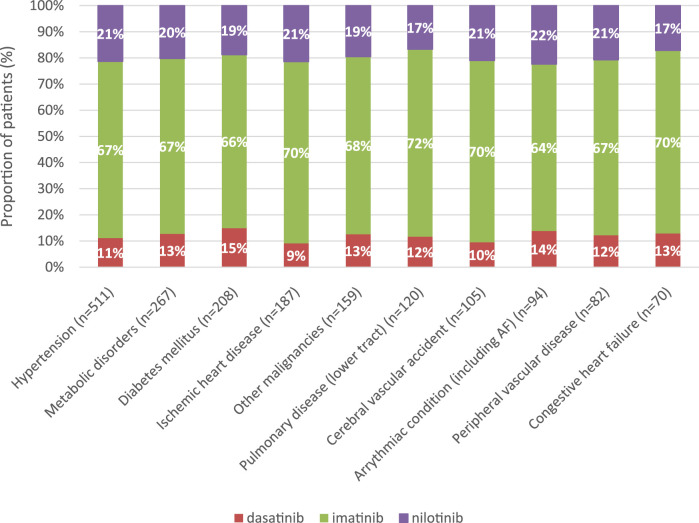
Distribution of TKIs according to the 10 most common comorbidities in first-line treatment of CML between 2011 and 2019. CML, chronic myeloid leukemia.

In all comorbidity groups, the majority of patients (67%–72%) received imatinib as first-line TKI treatment for CML. The second most common TKI was nilotinib (17%–22%), followed by dasatinib (9%–15%) (Figure 3).

### Overall survival of patients with CML

**FIGURE 4:**
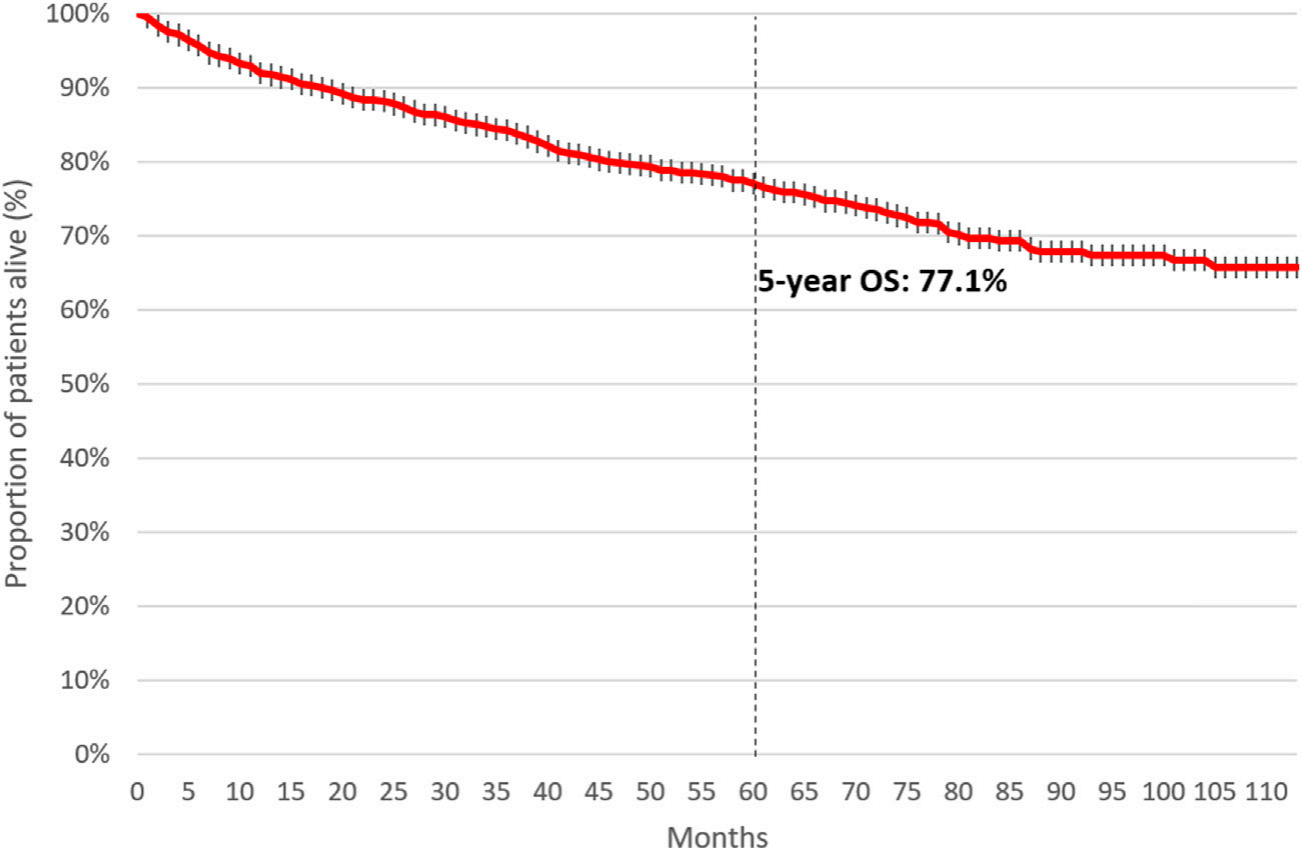
5-year overall survival of patients initiating first-line TKI treatment for CML between 2011 and June 2020. CML, chronic myeloid leukemia; TKI, tyrosine kinase inhibitor.

**FIGURE 5:**
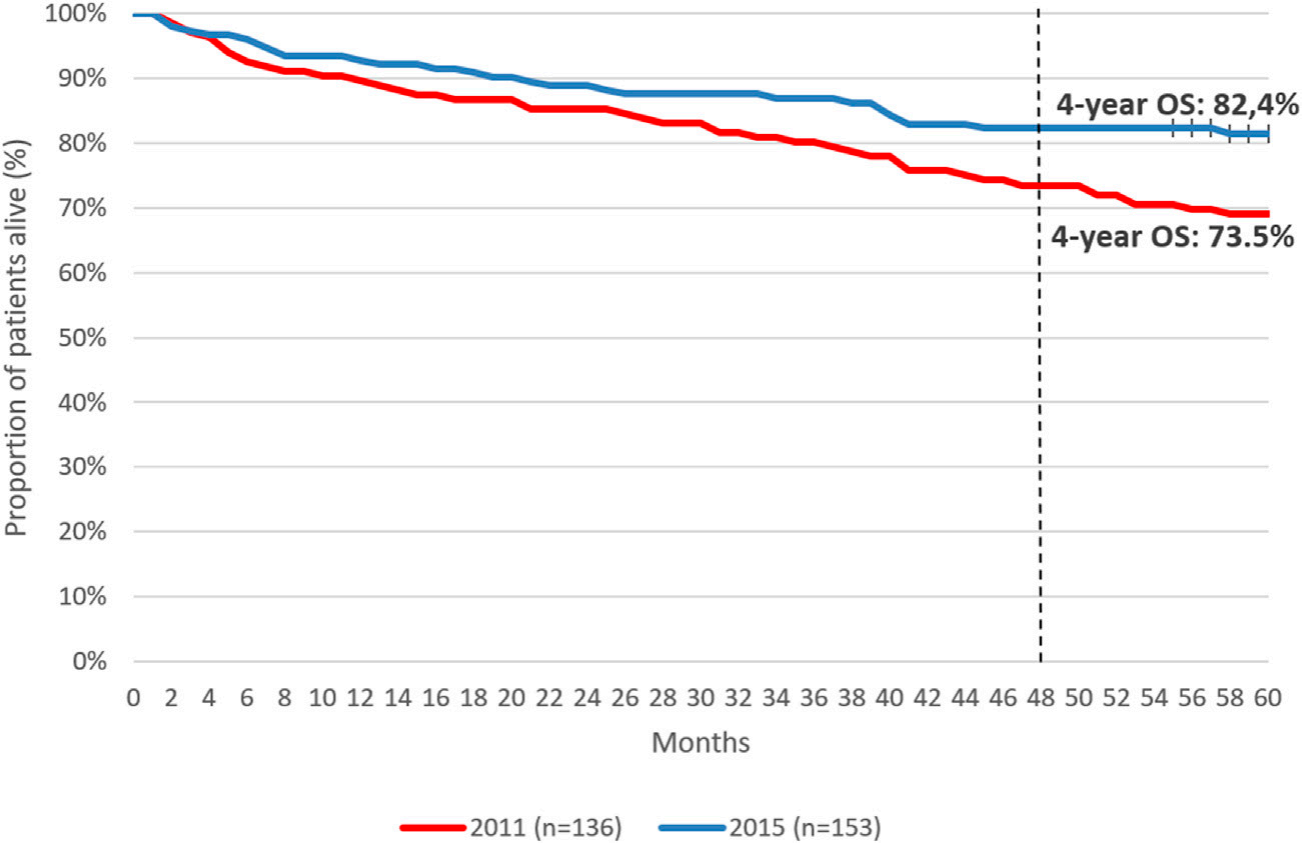
4-year overall survival of patients initiating first-line TKI therapy for the treatment of CML in 2011 vs. 2015. CML, chronic myeloid leukemia; OS, overall survival.

Five-year OS was 77.1% among patients initiating first-line therapy for CML. (Figure 4). Four-year OS was 73.5% and 82.4% in patients initiating first-line TKI treatment in 2011 and 2015, respectively (Figure 5, HR: 0.53 (95% CI: 0.32–0.87); *p* = 0.0118).

## Discussion

This nationwide, retrospective, longitudinal study examined comorbidities and treatment outcomes in an unselected real-world population of 1,407 patients with CML treated with TKIs over a 9-year period in Hungary. The number of patients initiating first-line TKI treatment for CML significantly increased between 2011 and 2019, with an annual average of 156 patients. The most common comorbidity was hypertension, and the proportion of patients with comorbidities was the lowest in the nilotinib and ponatinib treatment groups. Overall survival at 5 years was 77.1% among patients initiating first-line treatment for CML. 4-year OS was higher in those initiating TKI treatment in 2015 compared to patients starting TKI therapy in 2011 (82.4% and 73.5%, respectively).

Before the development of TKIs, CML was a fatal disease with a median OS of 5–7 years [21]. Treatment options for patients with CML included cytoreductive agents, interferon alpha (INF-α) and allogeneic stem cell transplantation (SCT) [22]. Imatinib, the first TKI to be approved in this setting in 2001, revolutionized the treatment landscape for CML and dramatically improved outcomes, transforming CML to a chronic disease with a close to normal expected life span. The pivotal International Randomized Study of Interferon and STI571 (IRIS) study compared imatinib 400 mg daily with IFN-alpha plus low-dose cytarabine in patients with newly diagnosed chronic-phase CML, and demonstrated the long-term, sustained efficacy and tolerability of imatinib with estimated 5-year and 10-year OS rates of 89% and 83.3%, respectively [23–25]. Further studies with CML patients under imatinib treatment reported 10-year relative survival rate of 92% [26].

However, approximately 30% of patients receiving imatinib as first-line treatment in the chronic phase might experience treatment failure either due to TKI resistance (primary or secondary) [27] or intolerance [28]. Inadequate treatment response due to imatinib resistance or treatment-related toxicities requires the change of TKI treatment [5]. These limitations of imatinib therapy initiated the development of the second- (dasatinib, nilotinib, bosutinib) and third-generation (ponatinib) TKIs which provide enhanced efficacy and activity against several imatinib-resistant BCR-ABL1 mutants [29–32].

The phase 3 DASISION trial demonstrated the safety and efficacy of dasatinib as first-line treatment in patients with CML in the chronic phase, with an estimated 5-year OS rate of 91% [29]. Frontline nilotinib 300 mg BID and 400 mg BID were associated with estimated 5-year OS rates of 93.7% and 96.2%, respectively, in the phase 3 ENESTnd study [30]. In the phase 3 BFORE trial comparing the efficacy and safety of bosutinib vs. imatinib in patients newly diagnosed with CP-CML, bosutinib was associated with a 5-year OS rate of 94.5% and showed superior efficacy vs. imatinib in terms of earlier and deeper molecular response [33]. Ponatinib was developed with the highest potency among all TKIs, and subsequently approved for the treatment of patients with CML who are resistant or intolerant to prior TKI therapies and in particular, in the presence of T315I mutation. In the international, randomized, phase 2 PACE study, evaluating patients resistant or intolerant to dasatinib or nilotinib, or who had the BCR-ABL1 T315I mutation regardless of prior TKI use, estimated 5-year OS was 73% in patients with chronic-phase CML [32].

Randomized controlled clinical trials almost never reliably represent everyday clinical practice. Patient selection often excludes elderly and frail patients with multiple comorbidities as well as patients with inappropriate ECOG (Eastern Cooperative Oncology Group) performance status. Despite the well-known limitations of real-world analyses, they can help to better understand the aspects of disease management that could be properly optimized to improve patient care. Real-world data may also help to guide the development of improved health plan operations, health system administration, cost management, epidemiologic research and can potentially contribute to the understanding and optimization of real-life treatment patterns and patient outcomes.

Since the advent of TKIs, the prognosis of CML is rather driven by coexisting comorbidities, than by CML itself [20]. Several studies have demonstrated the high prevalence of comorbidities among patients with CML treated with TKIs, and the strong association between comorbidities and outcomes [11, 34–36]. Specifically, in the randomized, 5-arm CML IV study of first-line imatinib therapy in patients with CML, higher Charlson Comorbidity Index (CCI) scores were significantly associated with lower OS, with an 8-year survival of 93.6% in patients with CCI = 2 and only 46.4% in patients with a score of >7 [20]. Of note, comorbidities had no negative impact on the success of imatinib therapy in terms of remission rates and disease progression, suggesting that patients with multiple comorbidities benefit from treatment with imatinib. Apart from overall survival, several studies suggest that comorbidities may also influence the risk of drug-related adverse events. A retrospective analysis of 125 chronic-phase CML patients treated with dasatinib at 21 Italian centers demonstrated a significant association between a higher comorbidity score and drug-related side effects [37]. Furthermore, pre-existing cardiovascular conditions have been shown to increase the risk of developing cardiovascular and arterio-occlusive events among CML patients treated with TKIs, especially in the case of newer agents [13, 38–40].

In this study, 53.5% of patients had at least one comorbidity at treatment initiation. The most common comorbidity of patients receiving any type of TKI in any treatment line was hypertension, which is in line with observations from other studies examining comorbidities among CML patients treated with TKIs in routine clinical practice. The ongoing, observational SIMPLICITY study is exploring the real-world use and management patterns of TKI in patients with chronic-phase CML receiving first‐line imatinib, dasatinib or nilotinib in the United States and 6 European countries [41]. Of 1,242 prospective patients enrolled between October 2010 and September 2015, 81% had baseline comorbidities, including hypertension (all TKIs: 36%; imatinib: 41%, dasatinib: 36%, nilotinib: 31%) [9]. In the EUTOS population-based registry of newly diagnosed CML patients in 20 countries, 55.5% of patients had baseline comorbidities, among which the most frequent was hypertension in 25.7% [11]. Age-standardized prevalence of hypertension among adults aged 30–79 years in 2019 was 48% (male: 56%, female: 41%) in Hungary [42] which is in line with our finding of 53,3% cases of hypertension, as the most common comorbidity among CML patients in Hungary.

Given the overlapping indications of approved TKIs and their different toxicity profiles, there is a need for guidance on the appropriate use of TKIs. While disease state, BCR/ABL1 TKD mutational status, additional chromosomal abnormalities and line of treatment are key considerations for the selection of TKI, patient comorbidities and differences in TKI safety profiles should also be taken into consideration. Nilotinib and ponatinib treatments are not recommended in patients with previous or concomitant cardiovascular disease due to the increased risk of cardiovascular complications [5, 43]. Dasatinib should be avoided among patients with respiratory failure and previous or concomitant pleuro-pulmonary disease [5]. Imatinib should be avoided in patients with significant renal impairment [5]. Nilotinib treatment may also be an increased risk in the presence of diabetes mellitus, hypertension, hyperlipidemia, and it is not recommended in case of previous pancreatitis [5]. Furthermore, patients treated with bosutinib may develop diarrhea, and treatment with bosutinib and nilotinib requires caution among patients with hepatic conditions [5]. Our findings regarding the selection of TKI for patients with patient profiles and comorbidities are in line with label recommendations. Dasatinib and nilotinib were typically prescribed for younger patients compared to imatinib, which is consistent with previous real-world observations showing that imatinib is predominantly prescribed for older patients [44]. An increasing proportion of elderly patients (65 years or older) was prescribed imatinib during the study period, which is likely to reflect the favorable tolerability of imatinib.

In this study, the 5-year OS of patients receiving first-line TKI treatment for CML was 77.1%, which is lower than OS rates reported by randomized trials and real-world publications. This may be attributed to the comorbidity profile of the Hungarian CML patient population. On the other hand, 4-year OS rates showed clinically relevant improvements during our study period: patients initiating first-line TKI therapy for CML in 2015 had a 4-year OS of 82.4%, compared to the 4-year OS of 73.4% in those initiating treatment in 2011. The improvement in OS rates may explained by the increasing knowledge and learning curve of hematologists on individual TKIs as well as the accumulation of real-world experience with TKIs and their adverse event profiles in patient populations with various comorbidities over time. As a growing body of evidence became available on TKIs over the past decade, hematologists were becoming more and more confident in the selection of individual agents while closely monitoring patient compliance. The OS improvement found in our study is a promising sign of improving patient management in real-world clinical practice and warrants further investigations to explore future trends in outcomes.

To our knowledge, our study is the first to describe the epidemiology, treatment patterns, and outcomes of patients with CML treated with TKIs in Hungarian clinical practice. However, it has certain limitations which need to be taken into consideration when interpreting the results. First, the NHIF database does not contain any information on non-reimbursed claims, laboratory data, patient parameters, symptoms, vital signs, adverse events, or the pathological and molecular features of CML. Furthermore, the database does not contain any data regarding the reason for a treatment switching, therefore, it did not allow for the evaluation of progression-free survival. Of note, overall survival was examined in our whole patient population and not separately for patients receiving different TKIs, therefore, conclusions regarding OS associated with individual TKIs cannot be drawn from this analysis.

## Conclusion

This study provides insights into the characteristics, treatment patterns, and outcomes of the Hungarian CML patient population treated with TKIs. The results show that the management of CML patients in Hungarian clinical practice is in line with international guidelines. The lower OS observed in this study compared to findings from other developed countries reflect the overall worse health status and comorbidity profile of the Hungarian patient population. Treatment patterns observed in routine clinical practice show that Hungarian caretaking hematologists adequately consider age, comorbidities, and other patient-related risk factors when selecting the proper TKI treatment for patients with CML.

## Data Availability

The original contributions presented in the study are included in the article/Supplementary Material, further inquiries can be directed to the corresponding author.
