# Corrigendum: Comorbidities and outcomes of patients with chronic myeloid leukemia treated with tyrosine kinase inhibitors: a real-world, nationwide, retrospective study from Hungary

**DOI:** 10.3389/pore.2024.1611758

**Published:** 2024-03-21

**Authors:** Peter Batar, Hussain Alizadeh, Gyorgy Rokszin, Zsolt Abonyi-Toth, Judit Demeter

**Affiliations:** ^1^ Department of Hematology, Faculty of Medicine, University of Debrecen, Debrecen, Hungary; ^2^ 1st Department of Medicine, Division of Haematology, Clinical Center, Medical School, University of Pecs, Pecs, Hungary; ^3^ RxTarget Ltd., Szolnok, Hungary; ^4^ Department of Biostatistics, University of Veterinary Medicine, Budapest, Hungary; ^5^ Department of Internal Medicine and Oncology, Faculty of Medicine, Semmelweis University, Budapest, Hungary

**Keywords:** chronic myeloid leukemia, real-word evidence, tyrosine kinase inhibitors, treatment outcome, overall survival

In the published article, there was an error in the **Funding** statement. It originally stated:

“The authors declare that this study received funding from Pfizer. The funder was not involved in the study design, collection, analysis, interpretation of data, the writing of this article or the decision to submit it for publication.”

The **Funding** statement has now been removed.

In the published article, there was an error in the **Conflict of interest** Statement. It originally stated:

“Authors GR and ZA-T were employed by RxTarget Ltd.

The remaining authors declare that the research was conducted in the absence of any commercial or financial relationships that could be construed as a potential conflict of interest.”

The correct **Conflict of interest** statement appears below.

“GR and ZA-T are employees of RxTarget Ltd., which was a paid contractor to Pfizer in connection with the development of this manuscript and was responsible for data curation and statistical analysis. This study was sponsored by Pfizer. Medical writing support was provided by Zsófia Barcza at Syntesia Medical Communications Ltd. and was funded by Pfizer. The funder had the following involvement in the study: administrative support and review regarding study design, statistical analysis, and manuscript development.

The remaining authors declare that the research was conducted in the absence of any commercial or financial relationships that could be construed as a potential conflict of interest.”

The authors apologize for these errors and state that this does not change the scientific conclusions of the article in any way. The original article has been updated.

