# Supplementary material for: Comorbidities and outcomes of patients with chronic myeloid leukemia treated with tyrosine kinase inhibitors: a real-world, nationwide, retrospective study from Hungary
Source: Pathol Oncol Res. 2024 Feb 20;30:1611497. doi: 10.3389/pore.2024.1611497 (PMC10913892; doi:10.3389/pore.2024.1611497)
Supplement: Supplementary file 1 [file DataSheet1.docx]

## Supplementary Material

**Supplementary Figure 1**

Distribution of patients treated with imatinib, dasatinib or nilotinib according to age (0–64 years vs.

≥65 years) between 2011 and 2019 in Hungary.

**Supplementary Figure 2**

Distribution of patients receiving imatinib, nilotinib, or dasatinib according to age (0–64 years vs.

≥65 years) between 2011 and 2019 in Hungary.

**Supplementary Table 1**

Predefined comorbidities and ICD codes defined based on conditions included in the Charlson Comorbidity Index (CCI)

AF: atrial fibrillation

**Supplementary Table 2**

Most common comorbidities of patients receiving any type of TKI for CML between 2011 and 2019 in Hungary, all treatment lines combined


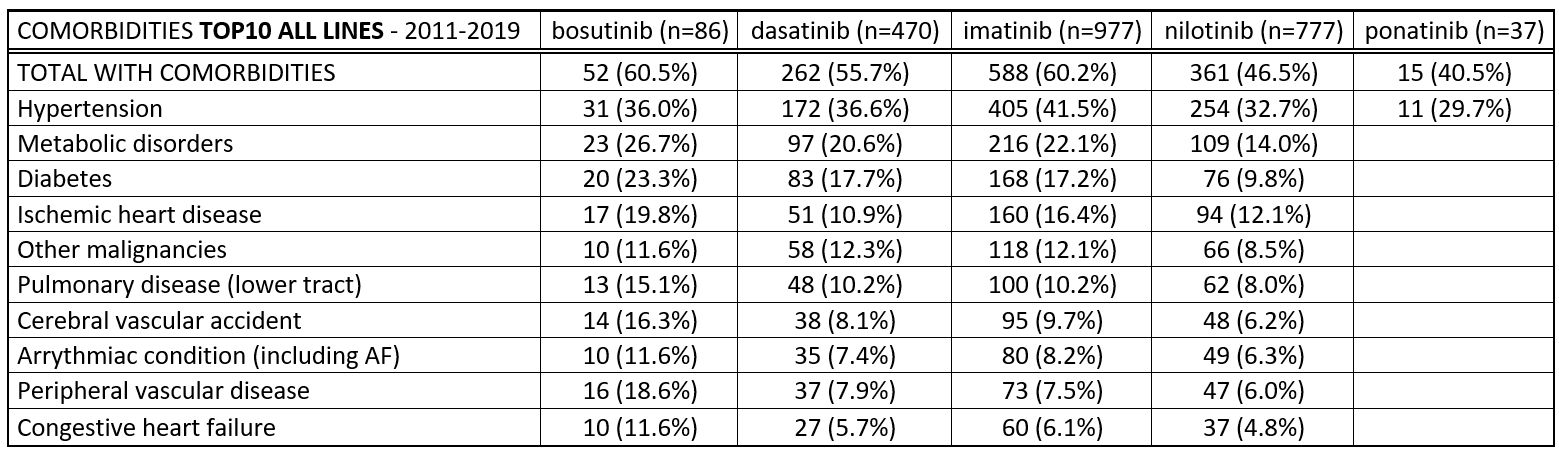


CML: chronic myeloid leukemia; TKI: tyrosine kinase inhibitor; AF: atrial fibrillation
